# Effectiveness of mRNA COVID-19 Vaccination on SARS-CoV-2 Infection and COVID-19 in Sicily over an Eight-Month Period

**DOI:** 10.3390/vaccines10030426

**Published:** 2022-03-11

**Authors:** Emanuele Amodio, Giuseppe Vella, Vincenzo Restivo, Alessandra Casuccio, Francesco Vitale, on behalf of the COVID-19 Surveillance Working Group of the University of Palermo

**Affiliations:** Department of Health Promotion, Mother and Child Care, Internal Medicine and Medical Specialties, University of Palermo, 90127 Piazza, Italy; giuseppe.vella02@unipa.it (G.V.); vincenzo.restivo@unipa.it (V.R.); alessandra.casuccio@unipa.it (A.C.); francesco.vitale@unipa.it (F.V.)

**Keywords:** COVID-19, vaccine effectiveness, mRNA vaccines

## Abstract

In order to reduce the spread of SARS-CoV-2 infection and the burden of disease, since 27 December 2020, Sicily has introduced a regional COVID-19 vaccination campaign. This study aimed at estimating the effectiveness of mRNA COVID-19 vaccines on SARS-CoV-2 infections and COVID-19. A retrospective cohort study was carried out on 3,966,976 Sicilian adults aged 18 years or more, who were followed-up from 1 January 2021 to 30 September 2021. The risk of SARS-CoV-2 infection, severe COVID-19, and COVID-19 death or intubation during the study period was compared among vaccinated with two mRNA doses and unvaccinated individuals. Cox regression, adjusted for age and sex, and a joint-point analysis on rate trends were performed. Overall, 2,469,320 (62.2%) subjects have been vaccinated and a total of 103,078 (2.6% of the entire population) SARS-CoV-2-positive subjects have been observed including 4693 (0.12%) severe COVID-19, 277 (0.01%) intubated, and 2649 (0.07%) deaths. After two months from vaccination, adjusted vaccine effectiveness was 81.3% against SARS-CoV-2 infection, 96.1% against severe COVID-19, and 93.4% against intubation/death. During the eight-month follow-up, statistically significant decreasing effectiveness trends were observed for all the evaluated outcomes (−4.76% per month against SARS-CoV-2 infection; −2.27% per month against severe COVID-19 and −2.26% per month against COVID-19 intubation/death). The study results confirm that mRNA COVID-19 vaccines have high real-world effectiveness, especially in the first months after vaccination. The vaccine effectiveness decreases over time and, even if the decrease is relatively small against severe outcomes, the increasing protection wane suggests the need for booster vaccination campaigns.

## 1. Introduction

Since the emergence of the novel coronavirus (SARS-CoV-2) infection in Wuhan, China, the epidemic has spread rapidly across countries worldwide [1]. According to the official statistics, in Italy from 30 January 2020 to 13 December 2021, 5,258,886 confirmed cases have been observed and 335,645 of them occurred in Sicily [2].

In order to reduce the spread of infection and the burden of disease, in Italy since 27 December 2020 anti-COVID-19 vaccines have been actively offered through a national vaccination campaign. The first vaccines available in Italy were both mRNAs: first Comirnaty (BNT162b2) and then Spikevax from 7 January 2021. The initial phase targeted front-line healthcare workers, recognizing this group’s particular high exposure and potential role in the transmission, and in a second phase, from February 2021, individuals at high risk of severe COVID-19, such as care home residents and their caregivers, people aged 80 years and over. From May 2021 vaccination was offered to the entire Italian general population.

A year later, as of 15 December 2021, over 106 million doses have been administered at a national level, granting about 46 million people a full cycle of vaccination with Comirnaty (69.8%), Spikevax (17.0%), Vaxzevria (10.8%), and Janssen (1.7%), respectively [3]. Overall, mRNA vaccines have shown more protection, followed by viral vector and inactivated vaccines [4].

Although several studies have confirmed the high/good effectiveness of mRNA vaccines, the spread of SARS-CoV-2 variants has raised serious concerns about the overall vaccine effectiveness against severe diseases caused by alpha, beta, and delta variants [5,6]. Moreover, a waning of the vaccine-induced immunity over time has been suggested to have a possible role in reducing effectiveness and increasing the SARS-CoV-2 transmission [7]. Both these factors have been associated with an increase in SARS-CoV-2 infections and COVID-19 hospitalizations of vaccinated subjects in several contexts as well as in Israel [8]. Since 27 September 2021, several countries, including Italy, have answered to these possible threats by campaigns to administer a third vaccine booster similar to Israel.

According to the previous considerations, since the vaccine’s impact on SARS-CoV-2 infections, COVID-19 cases and deaths could change from country to country, the international scientific community is strongly encouraged to perform long-term monitoring of the vaccine effectiveness through real-world data. 

This study aimed at estimating the effectiveness of mRNA COVID-19 vaccines against SARS-CoV-2 infections, severe COVID-19 cases, and COVID-19 deaths in a cohort of 3,966,976 Sicilian adults aged 18 years or more who were observed between January and September 2021.

## 2. Materials and Methods

### 2.1. Study Design and Participants

In this retrospective cohort study, we assessed the Sicilian population from 1 January 2021 to 30 September 2021. Sicily is the largest region in Italy and the most populous island of the Mediterranean Sea with a population of 4,833,705 inhabitants [9]. For the aims of the present study, we included in the analyses all individuals who met the following criteria:

- not infected by SARS-CoV-2 in 2020;
- aged ≥18 years;
- Sicilian residency.

According to the previous criteria, a total of 3,966,976 Sicilian individuals have been entered in the cohort on 1 January 2021 as unvaccinated and not previously positive to SARS-CoV-2. All demographic data have been obtained by the Italian National Statistical Institute (ISTAT) which is the official governative source of demographic data. 

Two other National electronics health registries were used to gather data for this study, all of them reporting data collected by the Sicilian Regional Health Office under the supervision of the Italian Ministry of Health. In detail, the first database, managed by the Italian National Institute of Health (ISS), included data about subjects who tested positive to SARS-CoV-2 using a PCR test, alongside demographic information. For each subject who tested positive to SARS-CoV-2, a standardized form was filled including sociodemographic variables (age, sex, and residency) and clinical information as date of the first positive test, hospitalization (occurrence and date), admission to intensive care unit, intubation (occurrence and date) and death (occurrence and date). SARS-CoV-2 positive subjects have been considered cases regardless of the presence of signs and/or symptoms, whereas in the case of presence of signs and/or symptoms they have been categorized as COVID-19 cases. According to the case definition provided by the Italian Minister of Health, COVID-19 cases have been considered severe cases when the clinical conditions of the patient required hospitalization or intubation or in case of death.

The second database included clinical information about the vaccinated cohort, the dates of each vaccine shot, and the vaccine type. Participants were considered vaccinated if they had completed a full vaccination cycle (two doses) with an mRNA vaccine and they have been followed-up from the month following the second dose vaccine administration.

Vaccination eligibility in Italy depended on the availability of doses and priority of protection. The first category that was vaccinated consisted of healthcare professionals, in order to avoid dangerous outbreaks in hospitals and assure health assistance to the population. They represented the cohort we observed from February to September, since most of them completed the vaccination cycle from December 2020 to February 2021. Then, month after month, vaccines were made available from the most vulnerable individuals to the least at-risk ones. Instead, the March–September cohort is mostly composed of extremely vulnerable individuals, because of age and/or comorbidities. The April and May cohort included every healthy person older than 50 years old, and, finally, the June and July cohort pertained to the general population of any age or condition.

All the previously reported databases have been merged by the Italian identification code, which is an alphanumeric code of 16 characters that unambiguously identifies individuals irrespective of citizenship or residency status.

The final database allowed us to characterize the cohorts of vaccinated and unvaccinated individuals and the time of event of SARS-CoV-2 positivity, severe COVID-19, and COVID-19 intubation/death. Each vaccinated cohort has been followed-up from the time of vaccination (from January to July) to the occurrence of the outcome or censoring.

### 2.2. Statistical Analysis

The description of the study population was carried out by calculating absolute and relative frequencies. The survival analysis was performed by evaluating the exposure person-time of the different cohorts of subjects. Specifically, vaccination status was categorized as a time-varying variable and all participants entered the cohort as unvaccinated. 

Individuals vaccinated with non-mRNA vaccines or subjects who only received one mRNA dose contributed to unvaccinated person-time before vaccination, according to some evidence suggesting that one dose of mRNA vaccine yields negligible protection [10]. The Kaplan–Meier estimator was used to construct survival plots. Hazard ratios (HR) and 95% CIs were calculated by Cox regression comparing rates of SARS-CoV-2 infection, COVID-19 severe cases, and COVID-19 death or intubation among vaccinated and unvaccinated individuals. A multivariable Cox regression analysis was performed by including age and sex as potential confounders. Vaccine effectiveness was calculated as 1-HR multiplied by 100. A Joinpoint analysis was used to evaluate the time trends of vaccine effectiveness, the direction and the intensity of the trend, and the monthly average percent change.

All the analyses have been performed by using R statistical software (version 4.0.3) and the statistical packages “metafor”, “survival”, “epiDisplay”, “ggplot”, “Segmented”, “Strucchange” and “ggfortify” [11]. 

## 3. Results

As reported in Table 1, a total of 3,966,976 Sicilian residents over 18 years (M:F ratio 0.93) have been observed from 1 January 2021 to 30 September 2021. Overall, 2,469,320 (62.2%) subjects have been vaccinated with two doses of mRNA vaccine and a large majority of them have been vaccinated between May and July 2021. During the follow-up period, a total of 103,078 (2.6% of the entire population) COVID-19 cases have been observed, with a higher incidence among subjects aged 18 to 30 years (3.67%) and 31 to 40 years (3.31%). Peaks of SARS-CoV-2 cases have been observed in February, March, and July (0.46%, 0.69%, and 0.86% of the at-risk population, respectively). Among SARS-CoV-2-positive subjects, 4681 (0.12%) were severe COVID-19 cases, 277 (0.007%) were intubated ones, and 2649 (0.07%) died while being infected.

**Figure 1:**
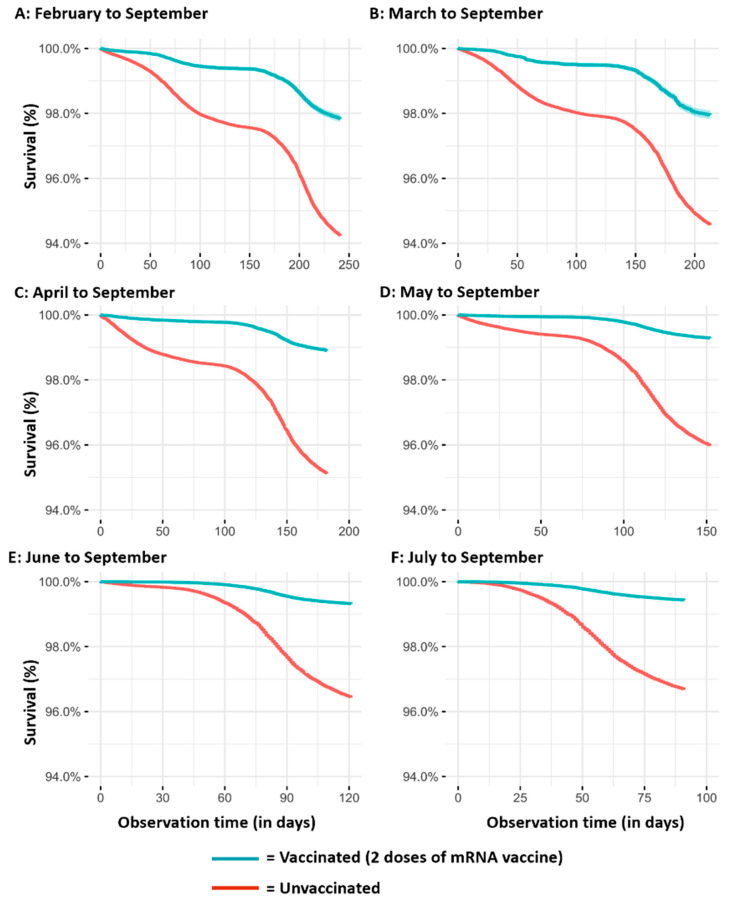
Kaplan–Meier survival analyses on SARS-CoV-2 infection over time in vaccinated and unvaccinated subjects according to the different observation periods ((**A**): February to September; (**B**) March to September; (**C**) April to September; (**D**) Mayto September; (**E**) June to September; (**F**) July to September).

**Figure 2:**
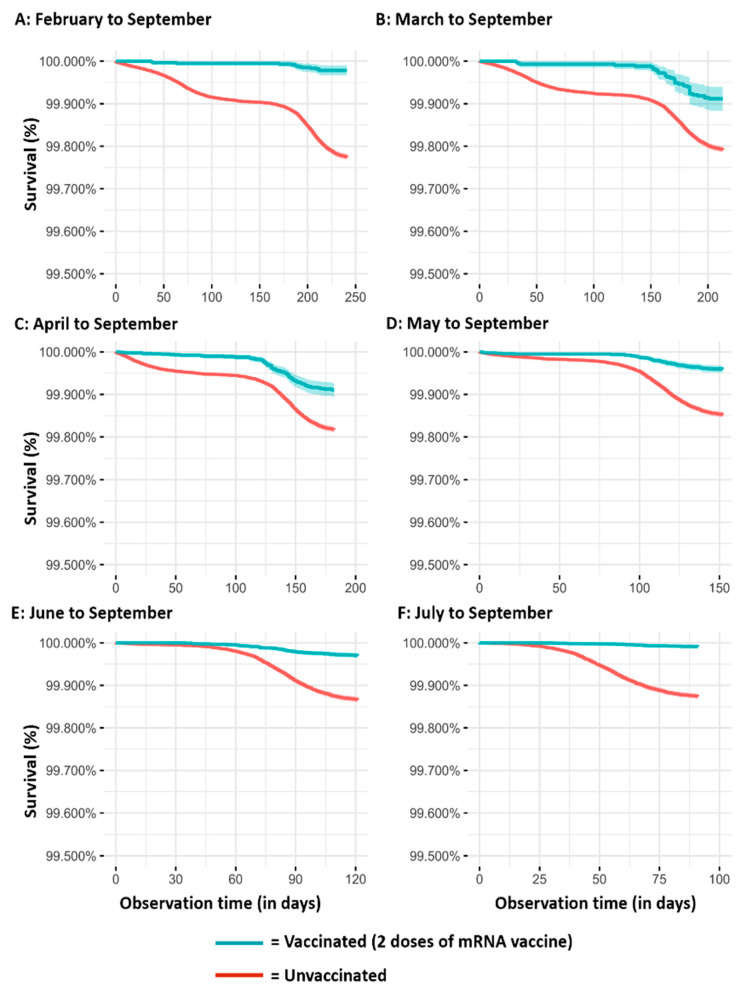
Kaplan–Meier survival analyses on COVID-19 severe cases over time in vaccinated and unvaccinated subjects according to the different observation periods ((**A**): February to September; (**B**) March to September; (**C**) April to September; (**D**) Mayto September; (**E**) June to September; (**F**) July to September).

**Figure 3:**
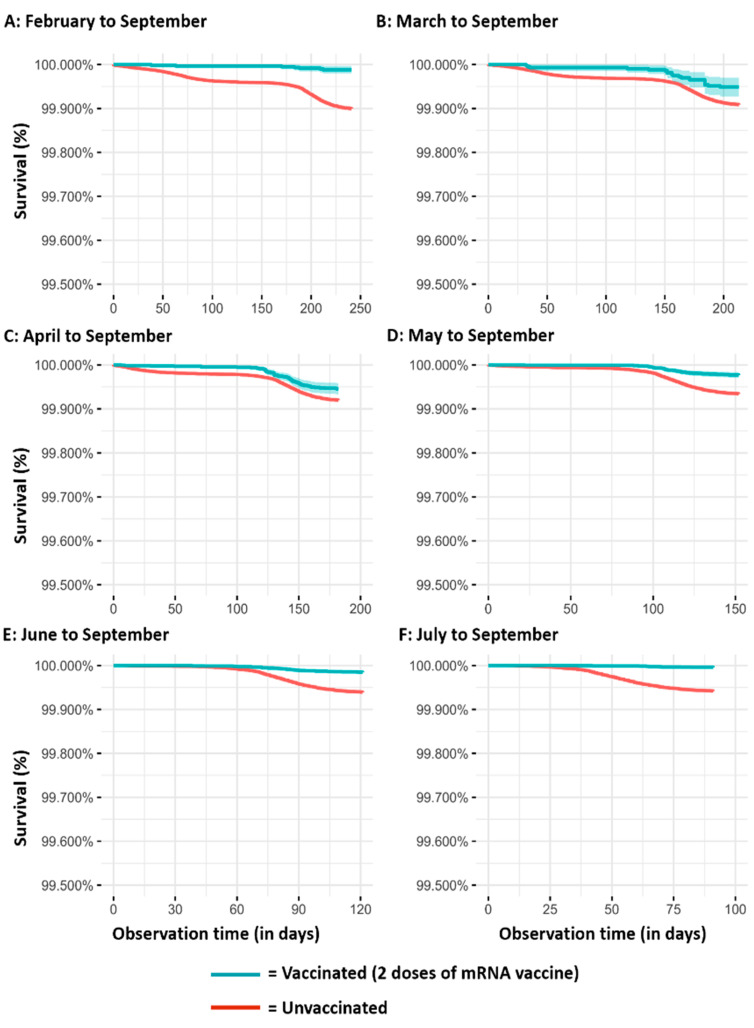
Kaplan–Meier survival analyses on COVID-19 intubation or death over time in vaccinated and unvaccinated subjects according to the different observation periods ((**A**): February to September; (**B**) March to September; (**C**) April to September; (**D**) Mayto September; (**E**) June to September; (**F**) July to September).

The results of the Kaplan–Meier survival analyses, according to the different observation periods, are reported in Figure 1 (SARS-CoV-2 infection), Figure 2 (severe COVID-19 cases), and Figure 3 (COVID-19 intubated cases or deaths). It is evident that regardless of the observation time periods (from three months in the cohorts observed from July to September to eight months in the cohorts observed from February to September), vaccinated subjects have a lower risk than those unvaccinated for all the assessed outcomes.

**Figure 4:**
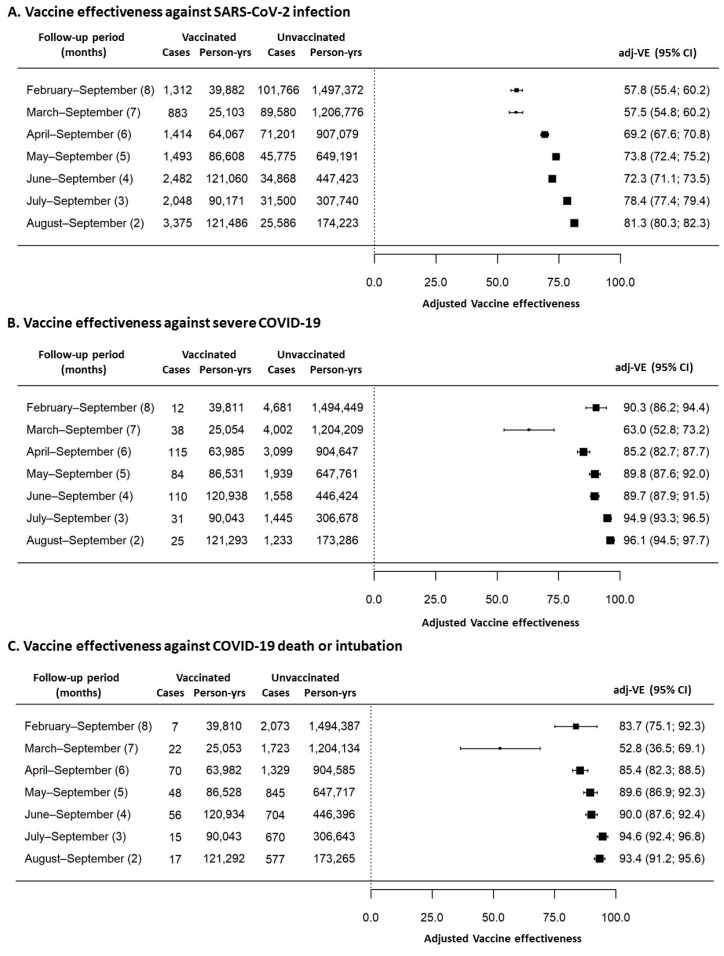
Vaccine effectiveness estimates after adjustment for age and sex according to the different assessed outcomes and follow-up periods. (**A**) Vaccine effectiveness against SARS-CoV-2 infection; (**B**) Vaccine effectiveness against severe COVID-19; (**C**) Effectiveness of vaccines against COVID-19 death or intubation.

Furthermore, Figure 4 summarizes vaccine effectiveness estimates, after adjustment for age and sex, according to the different outcomes and observation periods. Vaccine effectiveness against SARS-CoV-2 infections decreased from 81.3% (95% CI = 80.3–82.3%) in vaccinated subjects observed for two months (from August to September) to 57.8% (95% CI = 55.4–60.2%) in the cohort of vaccinated subjects observed for eight months (from February to September). Vaccine effectiveness against severe COVID-19 decreased from 96.1% (95% CI = 94.5–97.7%) in vaccinated subjects observed for two months (from August to September) to 90.3% (95% CI = 86.2–94.4%) in the cohort of vaccinated subjects observed for eight months (from February to September). Vaccine effectiveness against COVID-19 death or intubation decreased from 93.4% (95% CI = 91.2–95.6%) in vaccinated subjects observed for two months (from August to September) to 83.7% (95% CI = 75.1–92.3%) in the cohort of vaccinated subjects observed for eight months (from February to September).

Decreasing trends for vaccine effectiveness, measured as monthly percentage changes, were statistically significant for all the three evaluated outcomes (−4.76% per month, *p* < 0.001 against SARS-CoV-2 infection; −2.27% per month, *p* = 0.029 against severe COVID-19; −2.26% per month, *p* = 0.028 against COVID-19 intubation/death, respectively). 

## 4. Discussion

In the complex and ever-changing scenario that has characterized the development of the SARS-CoV-2 pandemic, vaccine effectiveness studies are producing evidence able to support resolute and effective decision making by governments. The evaluation of vaccine effectiveness during clinical trials was based on defined endpoints but it might not have reflected the impact of vaccination in the real world [12]. In this sense, it should be noted that clinical trials usually observe short time frames whereas, in the real world, time span could have a relevant impact on the intensity of vaccine protection. According to the study conducted by Cohn et al., vaccine effectiveness against infection declined over time for all vaccine types, going from 89.2% to 58.0% for mRNA-1273 (Spikevax/Moderna) and from 86.9% to 43.3% for BNT162b2 (Pfizer–BioNTech) in the 6 months of observation [13]. Pilishvili et al. found that vaccine effectiveness was estimated to be 94%, 14 days after a complete two-dose regimen of mRNA vaccines, analogous to clinical trials [14,15,16]. Chemaitelly et al. observed that vaccine effectiveness reached its peak at 77.5% (95% CI, 76.4 to 78.6) in the first month after the second dose and then it decreased over time [17].

Similar to previous studies, we have found a vaccine effectiveness against SARS-CoV-2 infection that declines from 81.3% (at month two) to 57.8% (at month eight), losing almost 5% of vaccine effectiveness per month. 

On the other hand, vaccine effectiveness trends that we have observed against severe COVID-19 outcomes showed some discontinuities in the first two observed cohorts (subjects vaccinated in January and in February, respectively). Intriguingly, individuals vaccinated in January maintained a very high vaccine effectiveness (90.3% and 83.7%) during the eight-month observation period. However, it should be considered that this first cohort was represented entirely by healthcare workers who are of workforce age, should be relatively healthy, and are usually more compliant to non-pharmacological prevention measures or pharmacological treatments. Thus, a higher vaccine effectiveness could be expected as a consequence of these previously reported confounding factors that we did not evaluate in our study. 

On the contrary, our second cohort, vaccinated in February, was represented by very frail individuals who, for clinical reasons, including immunosuppression, or being very elderly, were prioritized in vaccination access. The vaccine effectiveness observed in this latter cohort was significantly lower than that observed in all the other cohorts. This reduction in vaccine effectiveness was quite expected and it could be considered a direct consequence of their very frail condition and reduced immune protection, as already reported by others [18].

Aside from these two cohorts, overall vaccine effectiveness against severe COVID-19, death, or intubation showed steady declining trends over time with a reduction of about 2.7% per month.

Finally, the overall vaccine effectiveness, particularly as observed in the cohort of subjects vaccinated in July, does not appear to have suffered from the emergence of SARS-CoV-2 variant strains. In Italy, the predominant variant that affected our cohorts from January to June 2021 was the Alpha variant, with a brief appearance of the Gamma variant around June, whereas since July 2021 the Delta variant took its place as the most common strain (>80% of sequenced genomes) [19,20]. According to our data, considering cohorts vaccinated in June and July, we can argue that vaccination had a significant protective role also against Delta variant although we cannot estimate if this protection is lower than that against other variants.

The present study could suffer from some limitations. The first possible threat is that enrolled subjects were not checked for comorbidities or other major lifestyle risk factors (smoking habits, alcohol use, etc.) or testing patterns that, at an individual level, could differ between vaccinated and unvaccinated subjects and that could act as confounding factors. However, we suppose that vaccination coverage was increased among the at-higher risk of COVID-19 complications, including those with comorbidities, and thus the vaccine effectiveness observed in this study could underestimate the real protection effect. Moreover, as a second limitation, our considerations are set on the assumption that cellular immunity has no role and that new strains with comparable or greater risk are introduced regularly [21,22]. Unfortunately, this latter information about SARS-CoV-2 variants was not available to us and we cannot exclude vaccine effectiveness could change between different viral strains. Finally, the severity of COVID-19 has been recorded by different regional health professionals and, thus, a miss-classification bias could be not ruled out even if some guidelines have been distributed among Sicilian medical doctors in order to enhance data recording quality. Moreover, also a change in recording COVID-19 events due to different notification patterns over time could be not excluded as a potential source of bias.

## 5. Conclusions

Notwithstanding, we are confident that to date the present study is one of the most extensive carried out in a very large real-world cohort and, at the time of writing, includes a relatively long follow-up period. According to our findings, mRNA COVID-19 vaccines seem to have a very high real-world effectiveness, especially in the first months after vaccination. Furthermore, vaccine effectiveness decreases over time and, although this decrement is relatively minor against severe outcomes, the protection wane strongly supports the promotion and implementation of COVID-19 vaccination and booster vaccination campaigns.

## Figures and Tables

**Table 1:** Characteristics of the cohort of 3,966,976 Sicilian adults aged 18 years or more who were followed-up between January and September 2021.

		At Risk Population	Vaccinated Subjects (mRNA) Two Doses	SARS-CoV-2-Positive Subjects
		N (%, by Column Total)	N (%, by Column Total)	N (%, by Row Total)
Total		3,966,976 (100%)	2,469,320 (100%)	103,078 (2.6%)
Severe cases		3,966,976 (100%)	421 (0.017%)	4681 (0.12%)
Intubated cases		3,966,976 (100%)	12 (0.0005%)	277 (0.007%)
COVID-19 deaths		3,966,976 (100%)	257 (0.01%)	2649 (0.07%)
Sex				
	F	2,058,170 (51.9%)	1,301,789 (52.7%)	53,421 (2.6%)
	M	1,908,806 (48.1%)	1,167,531 (47.3%)	49,657 (2.6%)
Age				
	18 to 30 years	721,285 (18.2%)	434,735 (17.6%)	26,454 (3.7%)
	31 to 40 years	568,922 (14.3%)	322,386 (13.1%)	18,854 (3.3%)
	41 to 50 years	675,034 (17%)	409,610 (16.6%)	19,316 (2.9%)
	51 to 60 years	706,814 (17.8%)	447,447 (18.1%)	17,154 (2.4%)
	61 to 70 years	581,232 (14.7%)	356,749 (14.4%)	11,045 (1.9%)
	71 to 80 years	435,627 (11%)	291,117 (11.8%)	6083 (1.4%)
	81 to 90 years	235,643 (5.9%)	172,636 (7%)	3047 (1.3%)
	>90 years	42,419 (1.1%)	34,640 (1.4%)	1125 (2.7%)
Months				
	January	3,966,976 (100%)	60,769 (2.5%)	12,615 (0.3%)
	February	3,861,846 (97.3%)	43,253 (1.8%)	17,848 (0.5%)
	March	3,675,499 (92.7%)	128,867 (5.2%)	25,347 (0.7%)
	April	3,430,609 (86.5%)	208,372 (8.4%)	9918 (0.3%)
	May	3,092,272 (78%)	365,852 (14.8%)	3802 (0.1%)
	June	2,670,754 (67.3%)	362,224 (14.7%)	4587 (0.2%)
	July	2,299,112 (58%)	740,169 (30%)	19,738 (0.9%)
	August	1,538,086 (38.8%)	270,406 (11%)	263 (0.017%)
	September	1,267,070 (31.9%)	289,408 (11.7%)	8960 (0.7%)

## Data Availability

Data will be made available upon request to the corresponding author.
